# Carried over: Heat stress in the egg stage reduces subsequent performance in a butterfly

**DOI:** 10.1371/journal.pone.0180968

**Published:** 2017-07-14

**Authors:** Michael Klockmann, Friederike Kleinschmidt, Klaus Fischer

**Affiliations:** Zoological Institute and Museum, University of Greifswald, Greifswald, Germany; Oxford Brookes University, UNITED KINGDOM

## Abstract

Increasing heat stress caused by anthropogenic climate change may pose a substantial challenge to biodiversity due to associated detrimental effects on survival and reproduction. Therefore, heat tolerance has recently received substantial attention, but its variation throughout ontogeny and effects carried over from one developmental stage to another remained largely neglected. To explore to what extent stress experienced early in life affects later life stages, we here investigate effects of heat stress experienced in the egg stage throughout ontogeny in the tropical butterfly *Bicyclus anynana*. We found that detrimental effects of heat stress in the egg stage were detectable in hatchlings, larvae and even resulting adults, as evidenced by decreased survival, growth, and body mass. This study shows that even in holometabalous insects with discrete life stages effects of stress experienced early in life are carried over to later stages, substantially reducing subsequent fitness. We argue that such effects need to be considered when trying to forecast species responses to climate change.

## Introduction

Temperature is one of the most important ecological factors for ectothermic organisms, and the ability to cope with different temperatures is of key importance for species survival and distributions [1,2]. Exposure to high temperatures typically decreases individual fitness and does ultimately cause death [3,4]. Due to ongoing anthropogenic climate change, stressfully high temperatures will be more frequently encountered in the future, which may strongly affect biodiversity [5,6]. Increasing temperature extremes might be of particular importance here, as they have stronger effects on species distributions than mean temperatures [7,8]. Therefore, upper critical thermal limits have recently received substantial attention [9–11]. Here, tropical ectotherms may be particularly sensitive, as they live already close to their upper thermal limits [8,12–14].

When trying to assess the thermal tolerance of a given species, different aspects need to be considered. First, thermal tolerance may differ substantially throughout ontogeny, because developmental stages vary in size, morphology, physiology, and behaviour, which may easily affect thermal tolerance [11,15,16]. Second, life stages may not be entirely independent of one another, such that thermal stress experienced in a specific developmental stage may affect later life (“carry-over effects”; [17–21]). Third, even different generations may not be independent, being prone to transgenerational effects [22–24]. Nevertheless, the majority of studies on heat tolerance has focussed exclusively on a single stage, typically the adult one [13,25,26]. The concomitant neglect of other stages as well as potential carry over and transgenerational effects may lead to spurious results and misinterpretations [26–29], and it is therefore surprising that carry-over effects have not received more attention in the given context to date [20,21,30,31].

To add further complexity, negative effects of high temperatures on growth and development can also be important [14,32,33]. For instance, heat stress during development may reduce adult body size, which may in turn reduce subsequent heat tolerance [16,32,34–36]. Besides stress tolerance, body size may furthermore affect longevity, reproductive success as well as competitiveness [32,37–39], thereby potentially affecting even subsequent generations. Thus, enhancing our abilities to predict responses to climate change obviously requires the consideration of heat stress on survival and other fitness components throughout development and perhaps even across generations [19,25,40].

Against this background, we here investigate the effects of different temperatures experienced in the egg stage on the heat tolerance of later developmental stages in the tropical butterfly *Bicyclus anynana* (Butler 1897). Therefore, we test for heat survival (and growth) of hatchlings (i.e. shortly after temperature exposure), larvae and adult butterflies to test for the occurrence and extent of carry-over effects across developmental stages. We predicted that increasing heat stress perceived in the egg stage would reduce subsequent performance, but that detrimental effects are largely restricted to subsequent (i.e. larval) stages. We indeed found that heat stress experienced in the egg stage decreased subsequent survival, growth, and body mass, and that effects were detectable up to the adult stage. Given the holometabolic life cycle of butterflies, these results are surprising and exemplify the non-independence of developmental stages and the important role carry over effects may play in determining tolerance to environmental stressors.

## Materials and methods

### Study organism and egg sampling

*Bicyclus anynana* is a tropical, fruit-feeding butterfly ranging from southern Africa to Ethiopia [41]. The species inhabits a highly seasonal environment with alternating wet-warm and dry-cool seasons, such that it relies heavily on phenotypic plasticity to master associated challenges [42,43]. Temperature variation induces, for instance, plastic responses in wing coloration, growth and development, reproduction, and survival [14,16,44–47]. Reproduction is confined to the favorable wet season during which oviposition plants are abundantly available [48,49]. A laboratory stock population was established at Greifswald University, Germany, in 2008, from several hundred eggs derived from a well-established stock population at Leiden University, the Netherlands. Several hundred adults are used per generation to produce the subsequent generation, maintaining high levels of heterozygosity at neutral loci [50]. All animals were reared at 27°C, 70% relative humidity, and a photoperiod of L12:D12 within a single temperature-, light- and humidity-controlled climate chamber. To initiate the experiments, we sampled eggs from several hundred females. We did not use a family design here as previous work showed that family as compared with temperature effects are negligible [16].

### Experimental design

About 500 *B*. *anynana* females were allowed to oviposit on small maize plants, from which eggs were collected one day after oviposition. Eggs were placed into petri dishes in groups of 10 eggs per dish. Dishes were randomly divided among six groups, and afterwards exposed for 24 hours to 27 (control), 29, 31, 33, 35 or 37°C in climate cabinets (Sanyo MLR-351H; Bad Nenndorf, Germany). The climate cabinets were heated up to the target temperature before the transfer of petri dishes, thus, no ramping assay was used. All eggs were exposed to temperature treatments on day 2 after oviposition, and were kept at 27°C throughout except for the 24 h exposure time. The temperatures used are based on previous results, promoting strong differences in survival rates [16]. These temperatures are clearly within the range of temperatures experienced by *B*. *anynana* in its natural habitat [14], although exposure times are typically shorter than 24 h. Egg hatching success (mean per petri dish) was subsequently scored under control conditions (27°C, 70% relative humidity, and L12:D12 photoperiod). We used the percentage of dead individuals per dish for further analyses.

**Fig 1:**
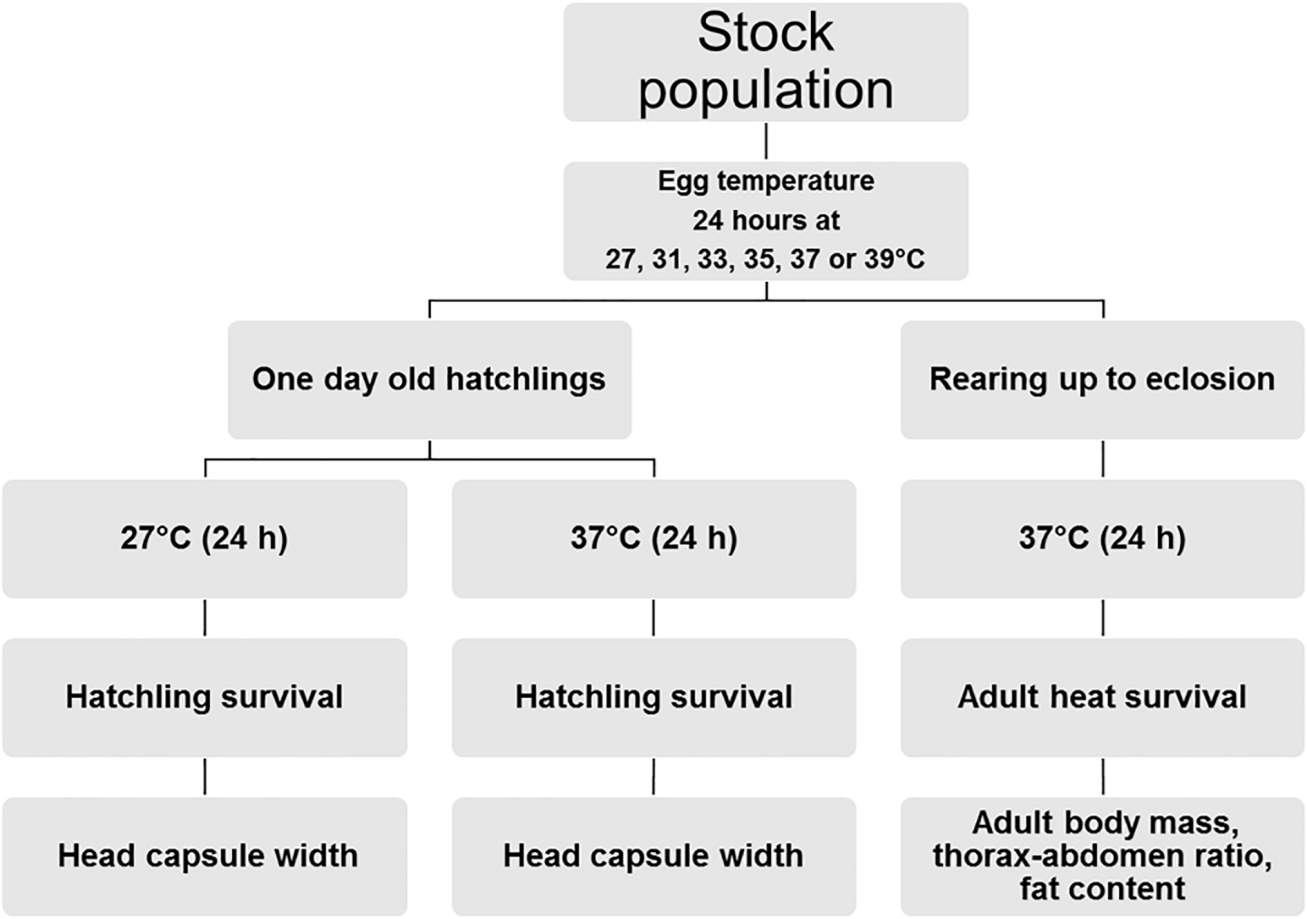
Schematic figure of the experimental design used. Eggs collected from stock population females were randomly divided and exposed to six temperatures for 24 hours each. Thereafter, one day-old hatchlings were exposed to either 27°C or 37°C, after which survival and head capsule width were measured. Another cohort of resulting hatchlings was reared under control conditions until adult eclosion and then exposed for 24 h to 37°C, after which heat survival and other traits were scored.

Resulting hatchlings were randomly divided into two cohorts (Fig 1). In the first part of the experiment, hatchlings were randomly divided and exposed for 24 h to either control (27°C) or heat (37°C) conditions (climate cabinets Sanyo MLR-351H; Bad Nenndorf, Germany). Therefore, hatchlings were transferred one day after hatching to petri dishes lined with moist tissue and fresh cuttings of their larval host plant (maize) in groups of 10 per dish. We used 23 to 55 replicates per egg temperature and stress treatment. Survival rate (in %) per petri dish was scored under control conditions 24 hours after exposure. Additionally we measured head capsule width of dead and alive hatchlings, using one individual per petri dish.

In the second part of the experiment, hatchlings were randomly divided among five cages per egg temperature with 30 individuals each, and were reared under control conditions until adult eclosion. Survival rates during larval and pupal development were scored per cage (%). One day after adult eclosion, all butterflies were individually transferred to plastic cups (125 ml) being provided with water, and exposed for 24 h to 37°C. Afterwards, individuals were back-transferred to control conditions. Note that all individuals were exposed to stressful conditions (37°C), as we were interested in the long-term effects of egg temperature on stress resistance. Survival rate was scored 24 h later (dead or alive). Then, all butterflies were frozen at -80°C. Thus, we here scored heat tolerance in adults having experienced different treatments exclusively in the egg stage. We measured adult body mass, thorax-abdomen ratio, and abdomen fat content for all butterflies after heat exposure. Therefore, frozen butterflies were first weighed to the nearest 0.01 mg (Sartorius LE225D). Afterwards legs, head, and wings were removed on dry ice, and the thorax and abdomen were separated and weighed. We calculated thorax-abdomen ratio as an indicator for the allocation trade-off between mobility (thorax) and reproduction (abdomen) [51]. Abdomen fat content, as an important indicator of condition, was measured after Fischer et al. (2003) but using the less poisonous acetone instead of dichloromethane [45]. In short, abdomens were weighed and subsequently dried for 48 h at 60°C. Abdomen dry masses were scored. Then, fat was extracted using acetone for 48 h, after which abdomens were once again dried and then weighed. Total fat content was calculated by subtracting the fat-free dry mass from the initial dry mass and is given as a percentage.

### Statistical analyses

We analysed (1) survival rates of eggs and hatchlings as the percentage of alive individuals per dish, (2) survival rates of larvae and pupae as the percentage of alive individuals per cage, and (3) variation in head capsule width, adult body mass, thorax-abdomen ratio, and abdomen fat content using general / generalized linear mixed models (GLMMs) with egg temperature, heat stress, and sex as fixed effects and cage as random effect (if applicable). Adult survival after heat stress was analysed using a nominal logistic regression on binary data (dead or alive) with egg temperature and sex as fixed effects, cage as a random effect (nested within egg temperature) and adult body mass, thorax-abdomen ratio, and fat content as covariates. Pair-wise comparisons after GLMs were performed employing Tukey’s HSD for unequal sample size. Throughout the text, means are given ± 1 SE. Data were analysed using STATISTICA 8.0 (StatSoft, Tulsa, OK, USA) or JMP 7.0.1 (SAS institute, Cary, NC, USA).

## Results

**Fig 2:**
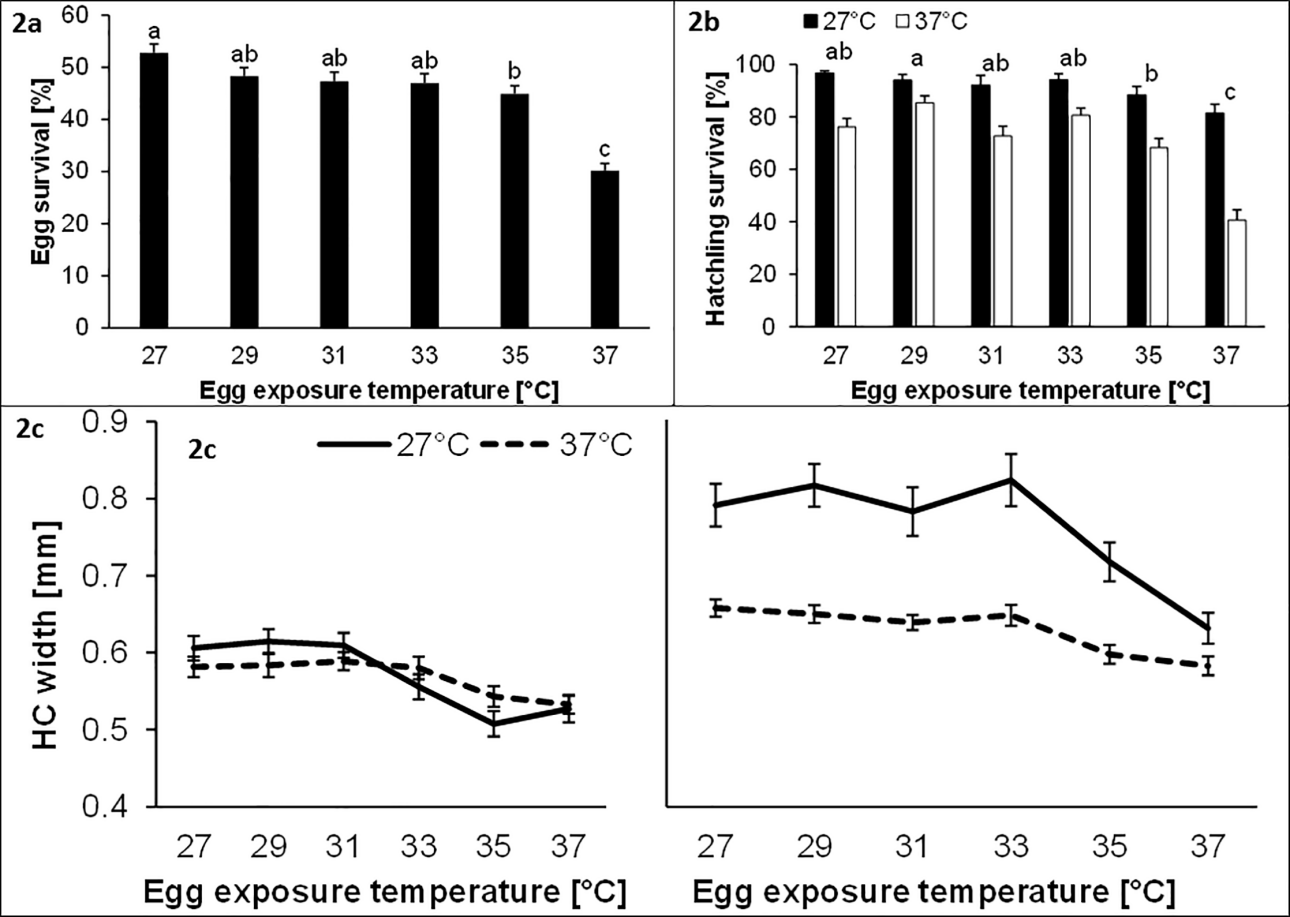
Egg survival rates in relation to temperature (a; 24 h at 27, 29, 31, 33, 35 or 37°C), hatchling survival rates in relation to egg temperature and heat stress (b; exposure of hatchlings for 24 h to 27°C or 37°C), and head capsule (HC) width in relation to egg temperature and heat stress for dead and alive individuals (c) in *Bicyclus anynana*. Given are means ± 1 SE. Sample sizes range between 132 and 199 groups (a), 23 and 55 groups (b), and 6 and 55 groups (c) with 10 individuals each. Different lower case letters above bars indicate significant differences among egg temperatures (Tukey’s HSD for unequal sample size).

**Fig 3:**
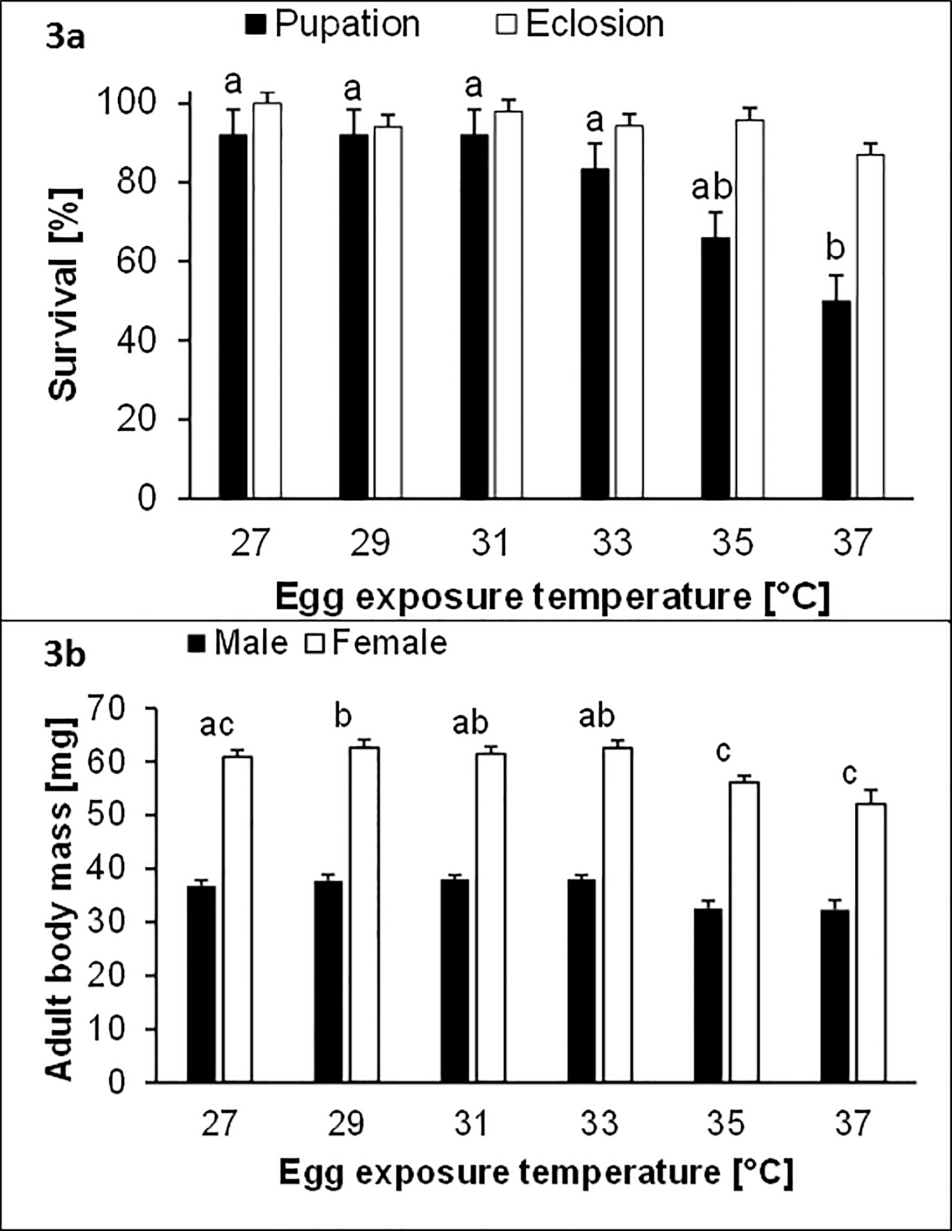
Survival rates until pupation and adult eclosion (a) and male and female adult body mass (b) in relation to egg temperature (24 h at 27, 29, 31, 33, 35 or 37°C) in *Bicyclus anynana*. Given are means ± 1 SE. Sample size were 5 cages with 30 individuals each (a) and 29 to 73 individuals each (b). Different lower case letters above bars indicate significant differences among temperatures (Tukey’s HSD for equal sample size).

Egg hatching success decreased significantly with increasing temperature from 52.8 ± 1.7% at 27°C to 30.2 ± 1.4% at 37°C (Table 1A; Fig 2A). Hatchling survival was significantly affected by both egg temperature and heat stress, being reduced at higher temperatures and following heat stress (control: 91.1 ± 1.7% > heat: 70.5 ± 1.2%; Table 1B; Fig 2B). Furthermore, a larger difference in hatchling survival between control and stress conditions was found at an egg temperature of 37°C (40.8%) compared with the other temperatures (8.6–20.4%; significant temperature x heat stress interaction). Hatchling head capsule width was significantly negatively affected by egg temperature and heat stress (control: 0.66 ± 0.007 mm > heat: 0.60 ± 0.004 mm; Table 1C; Fig 2C). Moreover, surviving hatchlings had significantly larger head capsule widths than dead individuals (alive: 0.69 ± 0.004 mm > dead: 0.57 ± 0.006 mm). However, all three main factors were involved in significant two-way interactions. First, effects of egg temperature on head capsule width were larger in control (reduction by 17.1% between 27 and 37°C) than in heat-stressed individuals (reduction by 10.0%; significant egg temperature x heat interaction). Second, effects of egg temperature were smaller in dead (reduction by 10.7% between 27 and 37°C) compared with surviving hatchlings (reduction by 16.2%; significant egg temperature x survival interaction). Third, effects of heat stress were restricted to surviving individuals (17.2% versus 0.3% difference between control and stressed individuals; significant heat x survival interaction). Data of the second cohort showed that egg temperature significantly affected survival rate during larval development while pupal development remained unaffected (Table 1D and 1E; Fig 3A).

**Table 1:** Results of general linear models (GLMs) for the effects of (a) egg temperature on egg survival rate, (b) egg temperature and larval heat stress on hatchling survival rate, (c) egg temperature, larval heat stress and survival (dead versus alive individuals) on head capsule width of hatchlings, and for the effects of egg temperature on the survival rate during (d) the larval and (e) the pupal stage in *Bicyclus anynana*. Significant *P*-values are given in bold.

**(a) Egg survival**	***MQ***	***DF***	***F***	***P***
Egg temperature	10957	5	27.2	**< 0.001**
Error	402	909		
**(b) Hatchling survival**	***MQ***	***DF***	***F***	***P***
Egg temperature	7239	5	16.3	**< 0.001**
Heat stress	43649	1	98.3	**< 0.001**
Temperature x Heat	1981	5	4.5	**< 0.001**
Error	444	456		
**(c) Head capsule width**	***MQ***	***DF***	***F***	***P***
Egg temperature	0.158	5	23.1	**< 0.001**
Heat stress	0.526	1	76.9	**< 0.001**
Survival	1.862	1	272.4	**< 0.001**
Temperature x Heat	0.019	5	2.8	**0.015**
Temp. x Survival	0.019	5	2.8	**0.017**
Heat x Survival	0.502	1	73.4	**< 0.001**
Temp. x Heat x Surv.	0.012	5	1.8	0.108
Error	0.007	728		
**(d) Pupation rate**	***MQ***	***DF***	***F***	***P***
Egg temperature	1536	5	7.4	**< 0.001**
Error	209	24		
**(e) Eclosion rate**	***MQ***	***DF***	***F***	***P***
Egg temperature	101	5	2.3	0.082
Error	45	24		

Adult survival after heat stress was only affected by adult body mass, being higher in surviving than in dead butterflies (49.8 ± 0.7 mg > 45.3 ± 1.1 mg). Adult survival did not differ among egg temperatures (Table 2). Butterflies differed significantly in adult body mass, which was lowest at 35°C and 37°C (Table 3A; Fig 3B). Additionally, adult mass was significantly higher in females than in males (females: 60.0 ± 0.6 mg > males: 36.5 ± 0.5 mg) and also differed between rearing cages. Thorax-abdomen ratio, in contrast, was significantly affected by sex only, being higher in males (57.3 ± 0.2%) than in females (43.0 ± 0.2%; Table 3B). Relative fat content, finally, differed significantly between males (16.3 ± 0.4%) and females (5.2 ± 0.2%) and among rearing cages (Table 3C).

**Table 2:** Results of a nominal logistic regression for the effects of egg temperature (fixed), cage (nested within temperature; random), sex (fixed), adult body mass, thorax-abdomen ratio and abdomen fat content (covariates) on adult heat survival in *Bicyclus anynana*. Significant *P*-values are given in bold.

Adult survival	*DF*	*F*	*P*
Egg temperature	5	5.52	0.356
Cage (Temperature)	24	34.86	0.071
Sex	1	1.78	0.182
Temperature x Sex	5	7.51	0.186
Adult body mass	1	10.72	**0.001**
Tho.-abd. ratio	1	0.04	0.841
Fat content	1	0.01	0.945

**Table 3:** Results of general linear mixed models (GLMMs) for the effects of egg temperature (fixed), cage (nested within temperature; random), and sex (fixed) on (a) adult body mass, (b) thorax-abdomen ratio, and (c) abdomen fat content in *Bicyclus anynana*. Significant *P*-values are given in bold.

**(a) Adult body mass**	***MQ***	***DF***	***F***	***P***
Egg temperature	0.0019	5, 25	4.2	**0.007**
Cage (Temperature)	0.0005	24, 649	5.3	**< 0.001**
Sex	0.0892	1, 649	1003.7	**< 0.001**
Temperature x Sex	0.0001	5, 649	0.2	0.967
Error	0.0001	649		
**(b) Thor.-abd. ratio**	***MQ***	***DF***	***F***	***P***
Egg temperature	42.7	5, 25	1.8	0.153
Cage (Temperature)	24.4	24, 647	1.4	0.108
Sex	30422.7	1, 647	1718.1	**< 0.001**
Temperature x Sex	32.9	5, 647	1.9	0.100
Error	17.7	647		
**(c) Fat content**	***MQ***	***DF***	***X***^***2***^	***P***
Egg temperature	1.4	5, 25	1.6	0.206
Cage (Temperature)	0.9	24, 626	2.2	**< 0.001**
Sex	219.7	1, 626	536.5	**< 0.001**
Temperature x Sex	0.1	5, 626	0.2	0.949
Error	0.4	626		

## Discussion

In line with earlier studies, we found strong negative effects of increasing temperatures on egg hatching success [16,52–57]. Such detrimental effects may arise, for instance, from denaturation of proteins, disruption of membrane structure, interactions with oxygen supply, or dehydration [55–57]. Similar considerations may apply to the increased mortality found in hatchlings exposed to heat stress (37°C). More interestingly, our results additionally demonstrate severe and long-lasting effects of thermal stress experienced early in ontogeny on later life. Specifically, we found that the temperatures exclusively experienced in the egg stage yielded effects on hatchling and larval survival resembling those found for egg hatching rate, and a similar tendency even for pupal survival. Thus, survival probability was clearly reduced in later life stages when having experienced temperature stress early in life. Especially in individuals that were exposed to heat stress twice (egg and hatchling stage) survival was compromised. Even in the adult stage, negative effects of higher egg temperatures were still visible, as adult mass was reduced in individuals having experienced higher egg temperatures. Reduced body mass may have detrimental effects on other fitness components such as stress tolerance and reproduction [32,34,58–60]. Thus, while egg temperature in our study had no direct effect on adult heat survival, our data suggest an indirect effect through reduced body mass.

The negative effects reported above suggest the existence of (energetic) costs associated with high temperatures. For instance, the heat shock response is considered to be costly which may reduce subsequent body size [56,61,62]. Small body size in turn often compromises other aspects of life such as stress tolerance, reproduction or competition [32,60]. Note here that higher temperatures experienced during development generally result in smaller bopdy size in ectotherms [16,32].

We measured hatchling head capsule width to also test for changes in body mass early in development. Note that head capsule width is closely related to hatchling mass in *B*. *anynana* [63]. Indeed, we found smaller head capsule widths in hatchlings that resulted from eggs exposed to higher temperatures [18,64,65]. This suggests increased metabolic losses at higher temperatures, which results in reduced body mass in turn contributing to the diminished overall performance. Alternatively, reduced feeding rate may have caused smaller head capsule widths [14]. Effects of egg temperature were larger in control than in heat-stressed hatchlings, likely reflecting the overall strongly reduced head capsule width in heat-stressed individuals (Fig 2C). Note that negative effects of heat stress were restricted to surviving individuals, indicating detrimental effects of heat on hatchling growth. Additionally, surviving hatchlings were larger than dead ones, meaning that larger individuals had a higher heat resistance [32,60] or that surviving individuals had more time for feeding and thus growth. The latter may also explain why the effects of egg temperature were more pronounced in surviving than in dead hatchlings. These results show that, as expected, acute heat stress has detrimental effects on hatchling size likely through negative effects on feeding and metabolism.

Taken together, the stress imposed on the egg stage was clearly visible throughout all subsequent life stages including resulting adults, being measurable as reduced body mass and survival. In holometabolous insects, the life cycle is clearly divided into distinct developmental stages (egg, larva, pupa, adult) separated by major developmental transitions. Potter et al. (2011), for instance, showed that negative effects of different egg temperatures were strong in early life but disappeared rapidly in subsequent life stages ([18]; see also [33]). Our results are somewhat similar in that effect size also declined with increasing time since the original stress. However, in our study effects were still visible even in the adult stage, indicating substantial carry-over effects throughout life and that developmental stages are not independent [18–21,31,64,66]. Furthermore, our results suggest that the stress and the concomitant deficiencies experienced early in life cannot be fully compensated for during development, although compensatory growth is a common feature in insects [21,67,68].

The females’ higher adult body mass compared with males is likely driven by a positive relation between body size and fecundity, while male insects are typically selected for fast development increasing mating opportunities [14,69]. Likewise, the males’ higher thorax-abdomen ratio and fat content seem to reflect sex-specific selection pressures, favouring flight ability and duration in males in order to succeed in competition for females [14,33,70].

Ongoing climate change, increasing ambient temperature and the frequency of extreme weather events like heat waves and drought periods, will probably have important consequences for life on earth [71,72]. Especially heat waves may have dramatic effects on population dynamics [72–74]. Our data show that even relatively short heat waves may have severe impacts based on (1) the direct mortality induced but (2) also through fitness reductions throughout the entire life cycle even if surviving the acute stress. Here, we demonstrated that heat stress during the egg stage reduced subsequent survival and body mass up to the adult stage. One caveat of our study is that we used an arbitrarily chosen time period of 24 hours to simulate heat weaves, which does obviously not resemble natural conditions particularly closely. However, based on earlier results we do not expect that more natural settings would change our conclusions substantially [75]. The shown detrimental effects even on adult body mass seem highly relevant for two reasons. First, body mass seems to be a crucial constraint on heat stress tolerance in *B*. *anynana* in general [16]. Second, this may even cause transgenerational effects [76], because smaller females typically lay fewer and/or smaller eggs potentially giving rise to offspring with reduced fitness [32,37–39,45]. In summary, our findings may have important implications for enhancing our abilities to predict the fate of particular species under ongoing climate change, indicating that carry-over effects throughout the life cycle as well as transgenerational effects need to be considered when trying to forecast species responses to climate change [19].
